# Factors associated with the likelihood of fall-related injury among people with lower limb loss

**DOI:** 10.1186/s40621-018-0171-x

**Published:** 2018-11-12

**Authors:** Stanford Chihuri, Christopher Kevin Wong

**Affiliations:** 1 Center for Injury Epidemiology and Prevention, Columbia University Medical Center, 722 West 168th St, Room 524, New York, NY 10032 USA; 2 Department of Anaesthesiology, College of Physicians and Surgeons, Columbia University, New York, NY USA; 3 Department of Rehabilitation and Regenerative Medicine, Columbia University Medical Center, New York, NY USA; 4 Program in Physical Therapy, Neurological Institute, 8th fl. 710 West 168th Street, New York, NY USA

**Keywords:** Amputation, Balance, Falls, Fall-related injury, Prostheses, Prosthetics, Amputee prognosis

## Abstract

**Background:**

People with lower limb loss that live in the community fall at a rate that exceeds that of other vulnerable populations such as hospitalized elderly people. Past research in a small single state study has identified factors associated with fall-related injury. The purpose of this study was to use a larger multistate sample of people with lower limb loss living in community settings to evaluate factors associated with fall-related injury in a multivariable model.

**Method:**

This retrospective cohort study included community-dwelling people with lower limb loss participating in wellness-walking programs in 6 states within the United States. Fall-related injury was considered injury sustained during a fall to the ground that required medical care. Pearson’s Chi-squared test and student’s t-test were used for descriptive statistics. Odds ratios and 95% confidence intervals from multivariable logistic regression modelling were used to estimate the likelihood of fall-related injuries.

**Results:**

Of the 303 subjects recruited, 257 (84.8%) were included in the analyses. Overall, 45 subjects (17.5%) reported at least a single fall-related injury. Most subjects reported two or more falls within the previous 12 months (*N* = 161, 63.1%), were male (*N* = 177, 68.9%), and were White (*N* = 212, 83.8%). Most falls were associated with gait (44.5%), activities of daily living (ADL, 15.7%), or ramps and/or stairs (12%). The likelihood of fall-related injury was elevated among females versus males (OR = 2.90, 95% CI 1.35, 6.24), people of non-White versus White race (OR = 4.79, 95% CI 1.06, 21.76), people with vascular amputations due to peripheral artery disease or diabetes versus non-vascular amputations (OR = 2.22, 95% CI 1.04, 4.73) and people with transtibial versus transfemoral amputations (OR = 2.32, 95% CI 1.01, 4.89).

**Discussion:**

Results of this study show that the likelihood of fall-related injury was significantly higher among women, non-White race, people with vascular and transtibial amputations. The results from this study were largely consistent with results from the prior multivariable fall-related injury model.

**Conclusion:**

The results highlight the association of female sex, non-White race, vascular and transtibial amputations with the likelihood of fall-related injury. Future studies may use the study findings to develop educational fall prevention programs for women, minorities, and people with vascular etiology and transtibial amputations.

## Introduction

Falls are a leading cause of injuries and injury mortality in the United States (US) and around the world (Bhattacharya et al. 2016; Centers for Disease Control and Prevention, National Center for Injury Prevention and Control 2016; Peden et al. 2002; Stevens et al. 2006). From 2001 to 2011 the estimated total cost of trauma-related inpatient care in the U.S. was $240.7 billion (Dimaggio et al. 2016). Although fall-related injuries affect all demographic groups, age-adjusted incidence of fall-related injuries has significantly increased 4% annually among older U.S women from 2004 to 2013 (Verma et al. 2006). While falls, fall-circumstances and fall-related injuries among older adults are well documented, there is less research in people specifically with amputations.

Previous studies with fewer than 50 subjects suggest a heightened risk of falls and fall-related injuries among people with lower limb (Centers for Disease Control and Prevention, National Center for Injury Prevention and Control 2016; Wong et al. 2015; Wong et al. 2016a). Falls have been associated with a fear of falling and lower levels of balance confidence among people with limb loss (Miller et al. 2001a) with decreased balance confidence associated with lower levels of prosthetic function (Wong et al. 2014). Falls, decreased confidence, and lower prosthetic function (Barnett et al. 2013). The personal, indirect, and non-medical costs incurred after fall-related injury may extend far beyond the $25,000 average estimated direct medical costs in the 6 months following a fall for 16 people with transfemoral amputations seen between 1987 and 2014 (Mundell et al. 2017).

More than 50% of people with lower limb loss who live in the community experience falls each year leading to an average fall-injury rate of 46.2 per 100,000 person-days which exceeds that of other vulnerable populations such as hospitalized elderly (Wong et al. 2015, 2016a). Among people with lower limb loss, medical inequities and lower health status are more frequent in women, people with vascular amputations, and non-White racial groups (Lefebvre and Lavery 2011; Dillingham et al. 2002). A previous study concluded that female sex, non-White race, vascular amputation, and age were significant factors associated with the likelihood of fall-related injury among people with limb loss (Wong et al. 2016a). However, the convenience sample was restricted to 41 subjects from New York state.

Overall only one study with more than 200 subjects has explored falls among community-dwelling people with limb loss; and no U.S. studies have included more than 50 subjects (Hunter et al. 2017). Incidence rates for falls and related injuries in the community ranging from 24 to 80% have been documented following amputation, with factors associated with falls varying across recovery stages in the 12 studies (Steinberg et al. 2018). Only a small subset evaluated factors associated with fall-related injuries among people living with limb loss in community settings (Wong et al. 2016a; Steinberg et al. 2018). Identifying people at high risk for fall-related injury from a larger population drawn from a broader cross-section of community-dwelling people living in the U.S. and the factors associated with the likelihood of fall-related injury may support development of future public health interventions for people with lower limb loss. The purpose of this retrospective study was to use a multistate U.S. sample of people with lower limb loss to evaluate the factors contributing to a multivariable model to identify people who had fall-related injury. A secondary purpose was to extract descriptive data regarding fall circumstances.

## Materials and methods

This retrospective cohort study included community dwelling people with lower limb loss participating in a wellness-walking program in six states (Indiana, Kentucky, Michigan, Maryland, New York and New Jersey). The community-based wellness-walking program was led by two former paralympians with lower limb loss and included lectures about exercise and physical activity, supportive discussion, and time for participants to walk. Consenting volunteers who participated in the wellness-walking program between January 2014 and December 2016 and walked using their prostheses with or without a walking aid were recruited. Excluded from the study were non-community dwelling program attendees who live within any medical care facility, or people who could not understand the study objectives or procedures due to English language barrier, or cognitive deficits. Also excluded were attendees who judged themselves to be unable to participate in the program due to cardiac or neurologic medical conditions, or who chose not to participate in the study data collection aspect of the wellness-walking program.

Subjects provided demographic information and amputation-related medical history i.e. self-report measures of balance confidence, prosthetic function, and fall history; and performed balance and gait speed assessments while using their prostheses. The study questionnaire included age, sex (male, female), race (White, Hispanic, African-American, Asian-American, American-Indian/Alaskan Native, or Hawiian/Pacific Islander) and state-level location; as well as medical history included amputation etiology and level. Amputation etiology was defined as vascular for peripheral artery disease and/or diabetes; and non-vascular for traumas, congenital abnormalities, and other medical issues such as cancer. The incidence of falls and fall-related injuries were recorded with a fall defined as an unexpected loss of balance resulting in the person on the ground. Fall circumstance in response to the question, “what were you doing when you fell” were recorded, such that any fall related to sliding on wet or slick surfaces was defined as a slips; any fall related to catching a foot on an obstacle such as curbs, stairs, leashes or things on the ground was defined as trip (Stevens et al. 2006). Fall-related injury was considered injury sustained during a fall and that required medical care by a doctor or emergency room staff, or required hospitalization or surgery. The study was conducted in adherence with the study protocol approved by the institutional review boards and ethical committees of participating university medical centers.

### Balance and gait measurements

The Activities-specific Balance Confidence (ABC) scale is a subjective assessment of confidence in the ability to maintain balance during 16 common activities such as reaching to a shelf, walking to a curb, negotiating icy sidewalks (Myers et al. 1998). The ABC scale has demonstrated test-retest reliability (ICC = 0.91), internal consistency (Cronbach’s alpha = 0.95), and moderate concurrent validity (*r* = .070) with functional gait measures in people with lower-limb amputation (Miller et al. 2003) with the internal consistency of each item on Rasch analysis of the 0–4 ordinal scale version used in this study ranging from 0.75–0.94 (Sakakibara et al. 2011). The ABC score has been associated with more prosthetic functional use (Wong et al. 2014) and a lower likelihood of a history of any fall (Wong et al. 2015). Prosthetic use and function was assessed with the Houghton Scale (HS) and mobility subscale of the Prosthetic Evaluation Questionnaire (PEQ). The 4-question HS quantifies daily prosthetic use and function in indoor and outdoor walking conditions (Houghton et al. 1992). Total scores range from 0 to 12 with higher scores indicating better function (Houghton et al. 1992) without appreciable ceiling or floor effects (Devlin et al. 2004; Wong et al. 2016b).

Although numeric HS scores have good to excellent test-retest reliability (ICC = 0.85–0.96) and moderate internal consistency (Cronbach’s alpha > 0.70) (Devlin et al. 2004) and concurrent validity (*r* > .62) with self-reported functional scales and performance-based walking ability measures (Wong et al. 2016b; Miller et al. 2001b), items like hours of wear that may vary for other reasons than functional ability and the non-parametric nature of the self-report scale may make score ranges more meaningful to report. Subjects were classified into the three HS score categories corresponding to distinct walking abilities; limited household walker (5 > HS), limited community/independent household walker (6 > HS > 8), and independent community walker (HS > 9) (Wong et al. 2016b). The 12-question mobility subscale of the PEQ (Legro et al. 1998) has demonstrated reliability in a multisite study (ICC = 0.85) (Resnik and Borgia 2011) and good person- and item-separation reliability (> 0.95) and construct validity (*r* = 0.78) on Rasch analysis for measuring mobility in both visual analog scale and 5-level response formats used in this study (Franchignoni et al. 2007).

Performance-based balance and gait was assessed using three selected Berg Balance Scale (BBS) tasks, and the Timed Up and Go (TUG) test and 2-min walk tests (2MWT) performed at self-selected pace. Program leaders gave consistent and detailed verbal and visual instructions for each measure. Physical therapists, prosthetists, and paraprofessionals in both fields completed the assessments and recorded results on standardized data collection forms. Because the wellness walking program allowed insufficient time to complete the entire BBS, three specific tasks were selected from the BBS; stand with eyes closed, look behind over shoulders, and turn 360 degrees. High inter -rater reliability of the 3 individual tasks with testers of varying clinical experience has been demonstrated in people with lower limb loss (ICC > 0.90) (Wong 2014). The tasks have been associated with falls (Wong et al. 2015) and range from easy to difficult to perform for people with lower limb amputation based on Rasch analysis of a comparable group of people with limb loss (Wong et al. 2013). The TUG and 2MWT were conducted on a premeasured walking path with subjects using their typical walking aids. For the TUG, individuals were required to stand up from a chair without using their arms, walk 3.05 m, turn around, walk back to the chair, and sit down safely. Performance times on the TUG for people using lower-limb prostheses have good reliability (*r* = 0.93–0.96) (Schoppen et al. 1999) and correlate with BBS scores (Melzer and Kurz 2009). The ability to rise from sitting without using hands correlates with body weight-adjusted lower limb strength (*r* > 0.54) (Bohannon et al. 2010) and provides insight into functional ability beyond the speed of timed performances (Melzer and Kurz 2009). The 2MWT, which required subjects to walk back and forth on a standardized 10 m level indoor path for 2 min, incorporated an endurance element to walking ability assessment and has demonstrated excellent inter-reliability (ICC = 0.98) and responsiveness to change in people using lower limb prostheses (Brooks et al. 2002).

### Statistical analysis

Only subjects with available data on fall-related injury i.e. injury sustained during a fall that required medical care by a doctor or emergency room staff, or required hospitalization or surgery were included in the statistical analyses. Subjects with available injury data were compared with eligible subjects who were excluded on the basis of missing fall-related injury to assess potential selection bias. Statistical power was calculated based on the potential number of variables in the model (*n* = 10) and the 1 variable to 10 subjects rule. Variables that were included in the final model included variables that were significant in bivariate models. For each variable, model assumptions were considered and evaluated. Assumptions that were evaluated included normality, heteroscedasticity, sample size and multicollinearity.

Descriptive statistics for demographic data (age, sex, race) and baseline clinical variables (falls in the previous 12 months, etiology, amputation level, Houghton score, ABC, TUG TMW and BBS scores) were computed. Differences between people who reported injuries and those that did not were assessed using Pearson’s Chi-squared test and the student’s t-test. Non-parametric tests (i.e. Fischer’s exact test and Mann Whitney U test) were used where relevant. Frequencies for self-reported fall circumstances and activities that subjects were doing when the fall occurred were graphed.

Simple logistic regression models were used in the bivariate analyses to compute crude odds ratios and 95% confidence intervals (CI). A multivariable logistic regression model was used to compute adjusted odds ratios and corresponding 95% CI. Odds ratios and 95% CI were used to estimate the likelihood of fall-related injuries. Statistical significance was set at *p* ≤ 0.05. All analyses were performed using SAS statistical software version 9.4 (SAS Institute, Inc.).

## Results

Of the 303 subjects recruited, 255 (84.8%) had available data on fall-related injury and were included in the analyses. Subjects with available injury data did not differ significantly in sex (*P* = 0.658), age (*P* = 0.2076), amputation etiology (*P* = 0.8207) and amputation level (*P* = 0.1801) to those with missing fall-related injury data. Excluded subjects (*N* = 47) were more likely to be non-White (18.0% vs. 6.6%, *P* = 0.010).

Overall, the majority of subjects reported two or more falls within the previous 12 months (*N* = 161, 63.1%), were male (*N* = 177, 68.9%), and were White (*N* = 212, 83.8%,). Few subjects (*N* = 45, 17.5%) reported at least a single fall-related injury requiring medical attention within the same time period (Table 1). The majority of those injured had transtibial amputations.

**Table 1:** Baseline demographic characteristics according to injury status during the study period

Characteristic	Total	Injury	No injury	*P* value
	*n* = 255	*n* = 44	*n* = 211	
	n (%)	n (%)	n (%)	
Age, years, mean(SD)	55.7 (15.2)	57.4 (11.7)	55.2 (16.0)	0.29
Sex				
Male	176 (69.0)	25 (56.8)	151 (71.6)	0.03
Female	79 (31.0)	19 (43.2)	60 (28.4)	
Race				
White	212 (83.1)	41 (93.2)	171 (81.0)	0.06
Non-White	41 (16.1)	3 (6.8)	38 (18.0)	
Etiology				
Vascular	112 (43.9)	24 (54.5)	88 (41.7)	0.30
Non-vascular	131 (51.4)	20 (45.5)	111 (52.6)	
Amputation level				
TFA/bilateral TFA	125 (49.0)	14 (31.8)	111 (52.6)	0.02
TTA/bilateral TTA	114 (44.7)	26 (59.1)	88 (41.7)	
Houghton score: ≥9	128 (50.2)	19 (43.2)	109 (51.7)	0.54
6–8	71 (27.8)	14 (31.8)	57 (27.0)	
0–5	43 (16.9)	9 (20.5)	34 (16.1)	
Falls in 12 months				
None	4 (1.6)	0 (0)	4 (1.9)	0.06
1	90 (35.3)	22 (50.0)	68 (32.2)	
≥ 2	161 (63.1)	22 (50.0)	139 (65.8)	
ABC, mean (SD)	32.6 (16.3)	30.7 (14.7)	33.0 (16.7)	0.35
TUG test, seconds, mean (SD)	11.3 (12.2)	13.1 (10.9)	11.0 (12.5)	0.48
TMW test, feet, mean (SD)	90.3 (45.3)	89.9 (41.7)	90.3 (46.1)	0.95
Berg Balance Scale items, mean (SD)	3.0 (1.2)	3.2 (1.0)	3.0 (1.3)	0.26

*TFA* transfemoral amputation, *TTA* transtibial amputation

**Fig. 1:**
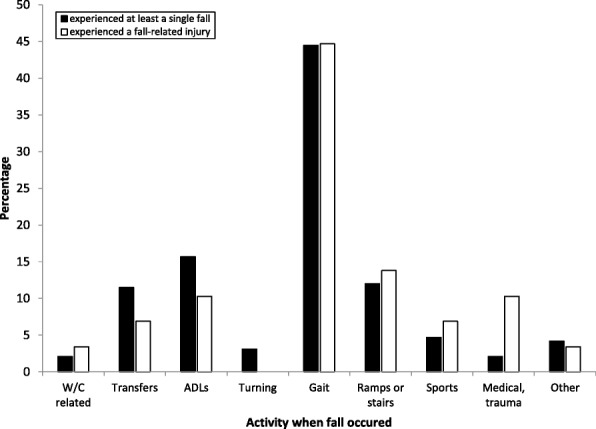
Activities when fall occurred among people with limb loss who experienced falls and those who experienced fall-related injuries. Black; Experienced at least a single fall. White; Experienced a fall-related injury. W/C: wheelchair; ADLs: activites of daily living

Among  subjects providing information on injuries, 191 (74.3%) reported the activities that led to falls. For most subjects, falls were associated with gait (*N* = 85, 44.7%) or specifically gait involving ramps or stairs (*N* = 26, 13.8%) (Fig. 1). There were no significant differences between injured and non-injured subjects with respect to performance-based clinical variables, including the BBS tasks, TUG, and the 2MWT test.

Table 2 shows the fall-related injury multivariable model that includes sex, race, amputation etiology, and amputation level (Hosmer and Lemeshow Goodness of Fit Chi-square = 2.95, *p* = 0.889). Results from the adjusted model show that females were almost three times as likely to have a fall-related injury compared to males (OR = 2.90, 95% CI 1.35, 6.24). Non-White people, who were comprised of African American, Hispanic and Other race were almost five times as likely to have a fall-related injury compared to White people (OR = 4.79, 95% CI 1.06, 21.76). People with vascular amputations were twice as likely to have a fall-related injury compared to those with non-vascular amputations (OR = 2.22, 95% CI 1.04, 4.73). Similarly, people with transtibial amputation were twice as likely to have a fall-related injury compared with those transfemoral amputations (OR = 2.32, 95% CI 1.01, 4.89).

**Table 2:** Crude and adjusted odds ratios and 95% confidence intervals from multivariable logistic regression model predicting fall-related injury

Characteristic	Unadjusted^a^OR	95%^a^CI	Adjusted ^a^OR	95%^a^CI
Sex:				
Female vs Male Sex	2.03	1.05–3.92	2.90	1.35–6.24
Race:				
Non-White vs White Race	3.04	0.89–10.3	4.79	1.06–21.76
Amputation Etiology:				
Vascular vs Non-vascular	1.41	0.74–2.69	2.22	1.04–4.73
Amputation Level:				
Transtibial vs Transfemoral	2.26	1.11–4.60	2.32	1.01–4.89
Age	1.01	0.99–1.03		
Timed Up and Go test	1.00	0.98–1.03		
ABC total score	0.82	0.53–1.22		
Houghton scale	0.94	0.84–1.05		
Balance (BBS 6,10,11)	1.08	0.83–1.41		

^a^*OR* odds ratio, *CI* confidence interval, *AUC* for adjusted model = 0.7

## Discussion

Results from this retrospective study show that female sex, non-White race, vascular and transtibial amputations were significant factors associated with the risk of fall-related injury. The findings were largely consistent with results from a prior, prospective single state survival analysis that found female sex (HR = 5.88, 95% CI 1.30, 26.5), non-White race (HR = 13.07, 95% CI 1.03, 165.76), and vascular amputation etiology (HR = 10.21, 95% CI 0.81, 129.09) to be strong predictors of fall-related injury (Wong et al. 2016a). Furthermore, no clinical variables were significant contributors to the multivariable model in both studies. However, the multivariable model derived from the current multi-state study was different from the prior study in two ways. First, the current study found age to be insignificant with age not included in the final model for fall-related injury. The prior study had included age as a covariate, albeit an insignificant one. The relationship between age and fall-related injuries may not be linear (Verma et al. 2006). For people with lower limb loss, activity may decline with age and people may participate in fewer activities that put them at risk of fall-related injury (Verma et al. 2006). Second, the retrospective study based on 257 subjects included amputation level in the multivariable model, while the smaller prospective study did not—although 8 of 11 people suffering fall-related injury had transtibial amputations (Wong et al. 2016a). While people with transtibial amputations achieve higher functional levels, they also engage in more activities that can lead to increased risk and more injurious falls (Wong et al. 2015; Jayakaran et al. 2014). This is illustrated by the fact that people with transtibial amputation, especially those that tend to fall more frequently, walk faster than those who do not fall (Vanicek et al. 2015).

The current study found no significant association between fall-related injury and all performance-based tests i.e. the BBS tasks, the TUG test, and 2MWT test. Figure 1 helps clarify this consistent, yet peculiar finding. While balance ability may be related to the incidence of falls (Wong et al. 2015), fall-related injury appears to be more related to individual characteristics and quite likely other factors or chance events not assessed in the study such as distractions and random occurrences. Among people injured, people who fell due to a trip were more likely to seek medical care from a doctor, and notable to be hospitalized or undergo surgery than people who fell due to a slip or a fall for other reasons. Tripping over carpets and rugs among the elderly is known to be a common risk that can lead to severe injury (Rosen et al. 2013). People with limb loss, have no tactile sensation to sense the presence of a rug or carpet against the prosthetic foot and may increase the risk of tripping falls. For individuals who catch a toe causing their knee to bend, a non-microprocessor enabled prosthetic knee will not provide resistance to collapse (Blumentritt et al. 2009). In addition, trips and slips cause different muscular demands that may be related to falls and injuries (Yang et al. 2007). In the current study, it was noted that people who slipped were able to walk faster than those who had falls for other reasons.

Figure 1 shows activities with increasing level of difficulty. People with limb loss who have poor balance and walking ability may engage in few challenging activities and therefore are at reduced risk for falls and injury. Although injuries still occur as shown in Fig. 1, for instance secondary to medical events including seizure, transient ischemic attack, and syncope, other trauma including a non-pedestrian motor vehicle accident, and accidents involving a Wheelchair (W/C) that was left unlocked and rolled off a curb, the number of falls and injuries is low. When balance and gait abilities are better, people feel more comfortable to engage in challenging activities such as going on ramps and sports. While fewer falls may occur in these activities compared to walking, the proportion of fall-related injuries was greater as shown in Fig. 1. In other words, the relationship between balance/walking ability and fall-related injury appears to be nonlinear with a yet to be explained or random component. While walking speed did not differ between people who did or did not report injuries, in our sample people who fell walked significantly slower than those who had not fallen. Among people who reported fall circumstance, however, people who slipped walked significantly (*p* = 0.0045) than those who had not.

Anecdotal findings regarding falls and injury highlight common risk factors for falls in the general population (Rosen et al. 2013) such as trips on rugs or falls involving curbs and stairs, slips on wet surfaces in the bathroom that were magnified for people with lower limb loss. Trips require rapid hip and knee flexion responses to lift the lower limb clear of any obstacle (Blumentritt et al. 2009). For people with lower limb loss, hip flexor and hamstring muscles generally atrophy and provide a weak response (Jaegers et al. 1995) while people who depend on prosthetic knees have no active knee flexion. Slips on wet/icy surfaces are also common and are known to pose challenges to people with lower limb loss whose gluteal muscles which are required for slip response (Yang et al. 2007) are typically weakened in people with lower limb loss (Raya et al. 2010). In addition, slips on sloping surfaces, falls on stairs, and turning motions were also among the most frequently reported circumstances and may be directly contributed to by limitations in prosthetic function: 1) Prosthetic feet provide limited ankle range of motion making adapting to sloping surfaces difficult (Burnfield et al. 2012); 2) Prosthetic knees generally provide no power to assist stair ascent and only independent community walkers typically qualify for hydraulic or pneumatic knees that assist in stair descent (Blumentritt et al. 2009) and 3) few prostheses allow rotational movement required for smooth turning motions and those that do provide approximately 1 degree of rotational motion (Su et al. 2010). All circumstances regarding falls were made riskier because the person with limb loss does not have the sensation to feel the risk occurring, such as a tightening dog leash.

This study has some notable limitations. First, while the broad sample recruited from multiple institutions in multiple states represents the typical age range of people with limb loss suggesting greater generalizability, the distribution of non-White participants and people with vascular disease was somewhat smaller than the population at large (Ziegler-Graham et al. 2008). Second, multiple testers pose a risk of unknown rater reliability. Most assessments were self-reports, however, and the performance-based walking assessments were timed tests that have been found to be reliable and easy to administer without complicated equipment or professional expertise (Schoppen et al. 1999). Nevertheless, the specific testers in this study were not tested for reliability performance measures included an abbreviated selection of BBS tasks was used to assess balance; a complete assessment may have yielded different results. In addition, the 2MWT and TUG walk way lengths differed from standard lengths, which may have affected performance. Third, the definition of fall-related injury in this study did not capture injuries for which self- or home-care was sufficient and thus may have underestimated the injury rate. For a person with limb loss, a simple abrasion or bruise to the amputated limb can limit prosthetic wear and functional ability and in the presence of diabetes or peripheral artery disease can lead to ulceration or subsequent reamputation (Wong et al. 2016b). Furthermore, the self-report questionnaire did not control for recall bias. Lastly, no attempt was made to quantify the magnitude or cost of any fall-related injury or document other injuries related to prosthetic use.

## Conclusion

Overall, the study highlights the association of female sex, non-White race, vascular and transtibial amputations with the likelihood of fall-related injury. Study results found no relationship among performance measures of balance and gait and injury incidence. Descriptive data showed slips and trips as common occurrences at the time of fall-related injury as in other populations; as well as the particular risk of negotiating ramps for people with limb loss. Women and minorities with vascular transtibial amputations may benefit from educational fall prevention programs.

## Abbreviations

2MWT: 2-Minute Walk Test slip
ABC: Activities-specific balance confidence
BBS: Berg balance scale
CI: Confidence intervals
HS: Houghton scale
OR: Odds ratio
PEQ: Prosthetic evaluation questionnaire
TUG: Timed up and go
